# Comparison of Cell Wall Polysaccharide Composition and Structure Between Strains of *Sporothrix schenckii* and *Sporothrix brasiliensis*

**DOI:** 10.3389/fmicb.2021.726958

**Published:** 2021-09-20

**Authors:** Héctor L. Villalobos-Duno, Laura A. Barreto, Álvaro Alvarez-Aular, Héctor M. Mora-Montes, Nancy E. Lozoya-Pérez, Bernardo Franco, Leila M. Lopes-Bezerra, Gustavo A. Niño-Vega

**Affiliations:** ^1^Laboratorio de Micología, Centro de Microbiología y Biología Celular, Instituto Venezolano de Investigaciones Científicas, Caracas, Venezuela; ^2^Instituto Superior de Formación Docente Salome Ureña, Santo Domingo, Dominican Republic; ^3^Laboratorio de Síntesis Orgánica y Productos Naturales, Centro de Química, Instituto Venezolano de Investigaciones Científicas, Caracas, Venezuela; ^4^División de Ciencias Naturales y Exactas, Departamento de Biología, Universidad de Guanajuato, Guanajuato, Mexico; ^5^Biomedical Institute, University of São Paulo, São Paulo, Brazil

**Keywords:** *Sporothrix* spp., fungal cell wall, beta-gucan, fungal virulence, Rhamnose, Rhamnomannan

## Abstract

*Sporothrix schenckii*, *Sporothrix brasiliensis*, and *Sporothrix globosa* are the main causative agents of sporotrichosis, a human subcutaneous mycosis. Differences in virulence patterns are associated with each species but remain largely uncharacterized. The *S. schenckii* and *S. brasiliensis* cell wall composition and virulence are influenced by the culturing media, with little or no influence on *S. globosa*. By keeping constant the culturing media, we compared the cell wall composition of three *S. schenckii* and two *S. brasiliensis* strains, previously described as presenting different virulence levels on a murine model of infection. The cell wall composition of the five *Sporothrix* spp. strains correlated with the biochemical composition of the cell wall previously reported for the species. However, the rhamnose-to-β-glucan ratio exhibits differences among strains, with an increase in cell wall rhamnose-to-β-glucan ratio as their virulence increased. This relationship can be expressed mathematically, which could be an important tool for the determination of virulence in *Sporothrix* spp. Also, structural differences in rhamnomannan were found, with longer side chains present in strains with lower virulence reported for both species here studied, adding insight to the importance of this polysaccharide in the pathogenic process of these fungi.

## Introduction

Sporotrichosis, a cutaneous and subcutaneous mycosis of humans and other mammals, is caused by species described within the pathogenic clade of the *Sporothrix* genus, of which *S. brasiliensis*, *S. schenckii*, and *S. globosa* are the three species of major clinical importance (de Beer et al., 2016). All species of the *Sporothrix* genus are thermo-dimorphic fungi, presenting a saprophytic sporulating mycelial phase at 25–28°C and a yeast-like pathogenic phase at 36–37°C. In humans, the disease is characterized by cutaneous and subcutaneous lesions with regional lymphocutaneous dissemination, although some pulmonary and systemic infections have been reported (Callens et al., 2006). It is a neglected infectious disease with a worldwide distribution, and a higher incidence in tropical and subtropical countries (Barros et al., 2011; Chakrabarti et al., 2014). The cutaneous disease begins with a traumatic inoculation of the fungus by contaminated soil or plant debris or through bites and scratches from infected cats (Barros et al., 2011; Chakrabarti et al., 2014). Multiple infections might arise from a single source, which can lead to outbreaks (Chakrabarti et al., 2014).

*Sporothrix schenckii* is the most widespread species of the pathogenic clade present in the Americas, Europe, Africa, and Asia and is mainly associated with a sapronosis (Zhang et al., 2015), similarly to *S. globosa*, which is predominant in Asia. Furthermore, *S. brasiliensis* is an emerging species related to cat-transmitted sporotrichosis, mainly described in Brazil but now, also, present in other South American countries (Chakrabarti et al., 2014; Etchecopaz et al., 2020; Rossow et al., 2020).

Differences in the virulence profiles in experimental models of infection have been reported within the pathogenic clade. *Sporothrix brasiliensis* is reported as the most virulent species, followed by *S. schenckii*, and *S. globosa*, with the latter been reported as the species with the lowest virulence of the three (Arrillaga-Moncrieff et al., 2009; Almeida-Paes et al., 2015; Clavijo-Giraldo et al., 2016; Lozoya-Pérez et al., 2020). However, differences within *S. schenckii* clinical isolates have also been reported, ranging from highly virulent to non-virulent isolates (Fernandes et al., 2013; Almeida-Paes et al., 2015). Some factors, such as melanization, thermotolerance, protein secretion, and immunogenicity have been related to the differences in virulence patterns between the *Sporothrix* spp. and within clinical isolates (Fernandes et al., 2013; Almeida-Paes et al., 2015).

The fungal cell wall protects the fungus, acting as an initial barrier against hostile environments while preserving the cell’s integrity against internal turgor pressure. It is a dynamic structure, presenting continuous changes in composition and structural organization as the cell grows or undergoes morphological changes (Latgé, 2007). These changes are strongly regulated during the cell cycle, or in response to environmental conditions, stress, and mutations in the cell wall biosynthetic processes (Klis et al., 2006; Ruiz-Herrera et al., 2006).

In general, fungal cell walls are bilayered structures, with the innermost layer comprising a core of covalently attached and branched β-(1,3) glucan, forming intrachain hydrogen bonds with chitin assembled into fibrous microfibrils, and all together forming a scaffold around the cell (Gow et al., 2017). The β-(1,3) glucan is a highly immunogenic molecule and is one of the main fungal pathogen-associated molecular patterns (PAMP) that bind to a very specific pathogen recognition receptor (PRR) present on the surface of the host’s immune cells, the C-type lectin dectin-1 (Hernández-Chávez et al., 2017). Chitin, is an important immunoreactive polysaccharide, that interacts with different PRRs in a size-dependent mechanism, where big (70–100μm) or very small (<2μm) chitin particles do not trigger immune reactions, while medium-sized chitin particles (40–70μm) induce a proinflammatory response, whereas small-sized chitin particles (2–10μm) trigger an anti-inflammatory response (Hernández-Chávez et al., 2017). In general, these two polysaccharides are often masked by the components of the cell wall outer layer, which differs from the inner scaffold layer (Erwig and Gow, 2016). The *S. schenckii* and *S. brasiliensis* cell wall is mainly composed of structural polysaccharides, β-glucans, and chitin and has a peptide-rhamnomannan (PRM) outermost layer (Lopes-Bezerra et al., 2018). More recently, it has been reported that the culture media have an influence on changes in the cell wall composition and structure, as well as on the virulence of *S. schenckii* and *S. brasiliensis* but not on *S. globosa* (Lozoya-Pérez et al., 2020).

Within the frame of all the previous bodies of evidence, in the present work, we examine and compare the *S. schenckii* and *S. brasiliensis* cell wall composition in different strains. The isolates studied here, showed distinct virulence profiles (Nascimento et al., 2008; Castro et al., 2013), and the analysis of possible differences in the composition and/or the relative content of cell wall components may add new important aspects that correlate with their difference in virulence profiles.

## Materials and Methods

### Strains and Growth Conditions

Fungal strains used in this study are listed in Table 1. The yeast morphology was obtained by growing cells on Brain Heart Infusion (BHI, Oxoid, Hampshire, United Kingdom) liquid medium, with continuous shaking at 100rpm for 4days at 37°C. Cells were inspected under a phase-contrast microscope (Nikon Optiphot, Japan) before being used to check for contamination or partial differentiation.

**Table 1:** Strains used in this work.

Organism	Strain	Virulence reported in the mouse model	Reference
*S. brasiliensis*	5110 (ATCC MYA 4823)	High	Castro et al., 2013
*S. brasiliensis*	IPEC 17943 (ATCC MYA 4824)	Low	
*S. schenckii*	15,383 (ATCC MYA 4820)	Mild	
*S. schenckii*	1,099-18 (ATCC MYA 4821)	Low	
*S. schenckii*	M-64 (ATCC MYA 4822)	Non-virulent	Nascimento et al., 2008

### Cell Wall Fractionation

Yeast cells from cultures in exponential phase were collected by centrifugation at 8,000 x *g* for 1h at 10°C. Briefly, the fungal pellets were suspended in distilled water with an equal volume of glass beads (0.45–0.50mm diameter) and shaken five times in a Braun homogenizer (Braun, Melsungen, Germany) for 1min, followed by 1min cooling on ice between shakings. Cell disruption was followed by light microscopy. Cell homogenates were washed out of glass beads with distilled water and centrifuged at 480×*g* for 5min at 4°C. The pellet was freeze-dried, weighted, and fractionated by alkaline separation (Previato et al., 1979; San-Blas and San-Blas, 1994; Lopes-Bezerra et al., 2018). Briefly, the freeze-dried material was re-suspended in 1M NaOH for 16h, and the suspension was centrifuged to separate the alkali-insoluble material from the supernatant (fraction 1). The supernatant was neutralized with 1N HCl, centrifuged and the pellet (alkali-soluble and acid-insoluble, fraction 2) separated from the supernatant (alkali and acid-soluble, fraction 3), which was further analyzed as described previously (Lopes-Bezerra et al., 2018). Rhamnomannan was obtained by treating fraction 3 with Fehling’s reagent at 4°C as reported previously (Previato et al., 1979). The insoluble copper complexes generated, were centrifuged, washed three times with 3% KOH, twice with neat ethanol, and collected. The resulting residue was suspended in distilled water and cations removed with Dowex 50W-X4 (H^+^ form; Sigma-Aldrich, St. Louis, MO, United States) for 1h at room temperature; the supernatant was precipitated by the addition of four volumes of neat ethanol. The residue was collected by centrifugation at 8,000×*g* for 10min (fraction 4, rhamnomannan). The mother liquor of the copper complexes was neutralized with acetic acid and centrifuged. The supernatant was dialyzed for 72h against distilled water and deionized with a mixture of Dowex 1 (HCO_3_^−^ form; Sigma-Aldrich, St. Louis, MO, United States) and Dowex 50W-X4 (H^+^ form), the filtrate was concentrated, and the polysaccharides present were precipitated by the addition of three volumes of neat ethanol (fraction 5). All fractions obtained were freeze-dried.

### Chemical Analyses of Cell Wall Fractions

Sugar and total amino acid content of cell wall fractions were determined as follows: for hexose content, 10mg of each cell wall fraction was resuspended in 1ml of 1M HCl, sealed in a 2ml Wheaton 176,776 ampoule, and heated for 3h at 100 °C. Hydrolyzed samples were diluted 1/10 or 1/100. Sugar quantification was accomplished by the Anthrone method for hexose content quantification in concentrated H_2_SO_4_. To determine amino acid and amino sugar contents, 10mg of each sample was resuspended in 1ml 6M HCl, sealed in a 2ml Wheaton 176,776 ampoule, and heated for 16h at 100 °C. Amino acid and amino sugar content were determined employing alanine and glucosamine solutions as standards, as described previously (Rondle and Morgan, 1955; Yemm et al., 1955). For rhamnose quantification, 10mg of fraction 3 was resuspended in 1ml of 1M HCl, sealed in a 2ml Wheaton 176,776 ampoule, and heated for 3h at 100 °C. Hydrolyzed samples were diluted 1/10 or 1/100. Quantification of methyl pentoses was conducted (Dische and Shettles, 1948) using 85.7% H_2_SO_4_ and 3% cysteine in the reaction mixtures and rhamnose to construct a standard curve.

### Infrared Spectroscopy

Samples were prepared as KBr pellets. IR spectra were recorded from 3,500 to 500cm^−1^, using a Nicolet iS10 IR spectrometer (Thermo Fisher Scientific, Waltham, MA, United States), coupled to the OMNIC 8.0 software, following the indications of the Infrared Spectroscopy Service, Center of Chemistry, IVIC, Caracas, Venezuela.

### Nuclear Magnetic Resonance Analysis

To obtain the structural data, ^13^C and ^1^H NMR were employed, briefly, samples of the polysaccharide fraction to be analyzed and standards (ca. 20mg) were solubilized in D_2_O or 2% NaOD and the spectra obtained at 75MHz with a recollecting time of 16h and 70°C using a Bruker 300 Ultrashield spectrometer, according to the indication of the Nuclear Magnetic Resonance Service, Center of Chemistry, IVIC, Caracas, Venezuela.

### Analysis of Chitin Exposure on the Cell Wall Surface Using Flow Cytometry Analysis

For chitin exposure analysis, cells were stained with 1mg/ml wheat germ agglutinin-fluorescein isothiocyanate (Sigma-Aldrich, St. Louis, MO, United States), for 60min at room temperature. Flow cytometry was performed in a MoFlo XDP apparatus (Beckman Coulter), collecting 50,000 singlet events. Fluorescence of positive events was recovered from the compensated FL3 (green) channel using unlabeled yeast cells. Total population densities were gated and analyzed using FlowJo (version 10.0.7) software.

The heat-killed (HK) cells were prepared by incubating at 60°C for 2h. The cellular death was confirmed by incubating aliquots of the preparations in YPD plates at 37°C for 5days.

### Statistical Analysis

Quantifications of cell wall components were made by triplicate. Statistical analyses were done by the Tukey Honestly Significant Difference (HSD) *post hoc* test. Differences were considered statistically significant at *p*<0.05.

## Results

### Cell Wall Composition and Structure of Sporothrix Strains Under Study

**Figure 1:**
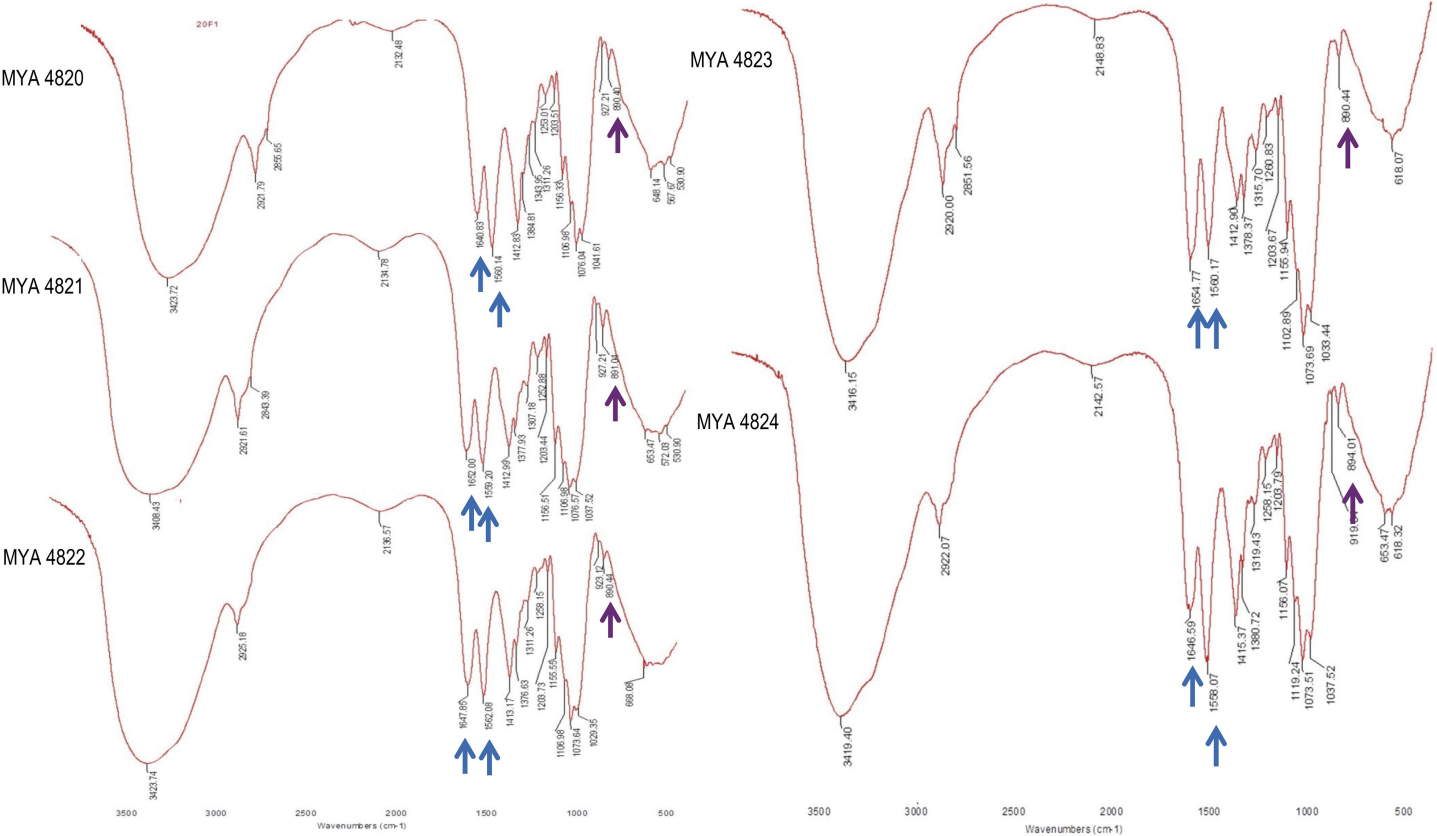
*Sporothrix schenckii* strains IR spectra of alkali-insoluble polysaccharides. Here, the signals corresponding to all strains are shown. Chitin and β-glucan signals are indicated with blue and purple arrows respectively, also are present signals of β-glucans (1,3 and 1,6 evidenced by peaks at 1156, 1076, and 1,041cm^−1^).

Structural and chemical analyses of polysaccharides from yeast walls of *S. schenckii* strains MYA 4820, MYA 4821, MYA 4822, and *S. brasiliensis* strains MYA 4823 and MYA 4824 were analyzed (Table 1). Cell walls from BHI-grown cells were purified and fractioned by the acid and alkali solubility and insolubility methods, as previously reported for *Sporothrix* cell wall analyses (Previato et al., 1979; Lopes-Bezerra et al., 2018). For polysaccharide structural characterization, IR spectroscopies, as well as proton and ^13^C nuclear magnetic resonance (^1^H-NMR and ^13^C-NMR respectively) were used, and the generated spectra compared with IR, ^1^H-NMR, and ^13^C-NMR spectra previously reported for *S*. *schenckii* (Travassos et al., 1973; Gorin et al., 1977; Gow et al., 1987; Lopes-Alves et al., 1992; Lopes-Bezerra et al., 2018). For the five strains analyzed, IR spectra of the alkali-insoluble cell wall fraction showed characteristic polysaccharide absorption signals (Figure 1), showing a strong and wideband around 3,400cm^1^ and additional bands around 2,921, 1,641, and 1,412cm^−1^ (Rodríguez-Brito et al., 2010). Absorption bands around 1,557 and 1,662cm^−1^ evidenced the presence of chitin, while β-glucan is evidenced by absorption bands at around 897 and 1,378cm^−1^ (Rodríguez-Brito et al., 2010). Also, the presence of absorption peaks belonging to β-(1,3)-(1,6)-glucan (1,160, 1,078, and 1,044cm^−1^; Synytsya and Novak, 2014), is present in all the IR spectra obtained from all the strains.

**Figure 2:**
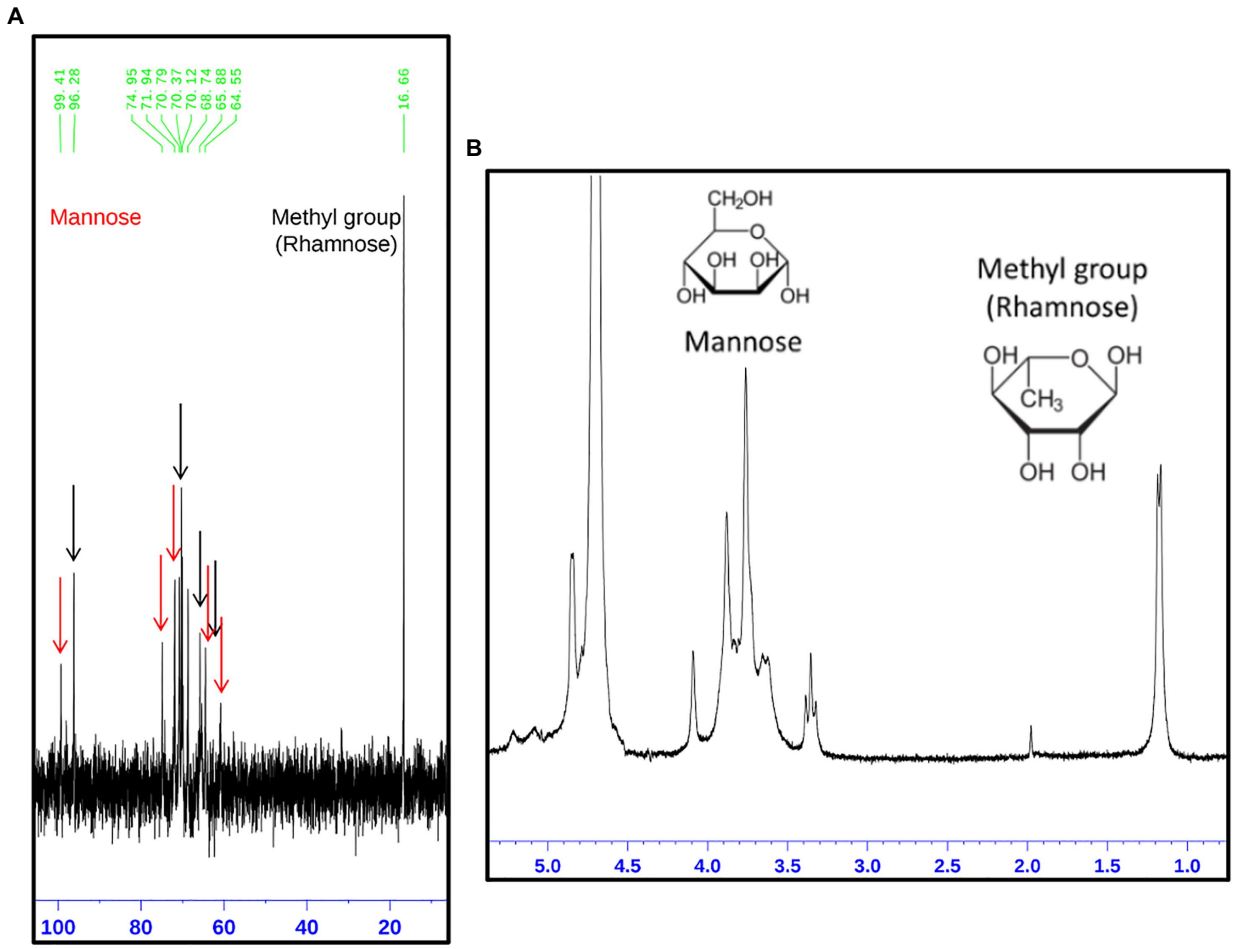
Structural analysis of the rhamnomannan present in the *Sporothrix* strains employing ^13^C-NMR and ^1^H-NMR (**A**,**B**, respectively). On the image, the spectrum corresponding to the rhamnomannan fraction of *S. schenckii* strain MYA 4820 is presented as a representative spectrum of both, *S. schenckii* and *S. brasiliensis* strains under study. **(A)** The signals corresponding to the carbon atoms in the mannose and rhamnose residues are shown as arrows, red for mannose and black for rhamnose. The corresponding signals are shown in Table 1. **(B)** Show the presence of the methyl group belonging to rhamnose (1.18 and 1.17p.p.m.) and the signals correspond to the proton bound to carbon 5 next to the methylene group for carbon 6 in the mannose ring (3.32, 3.35, and 3.39p.p.m.).

**Figure 3:**
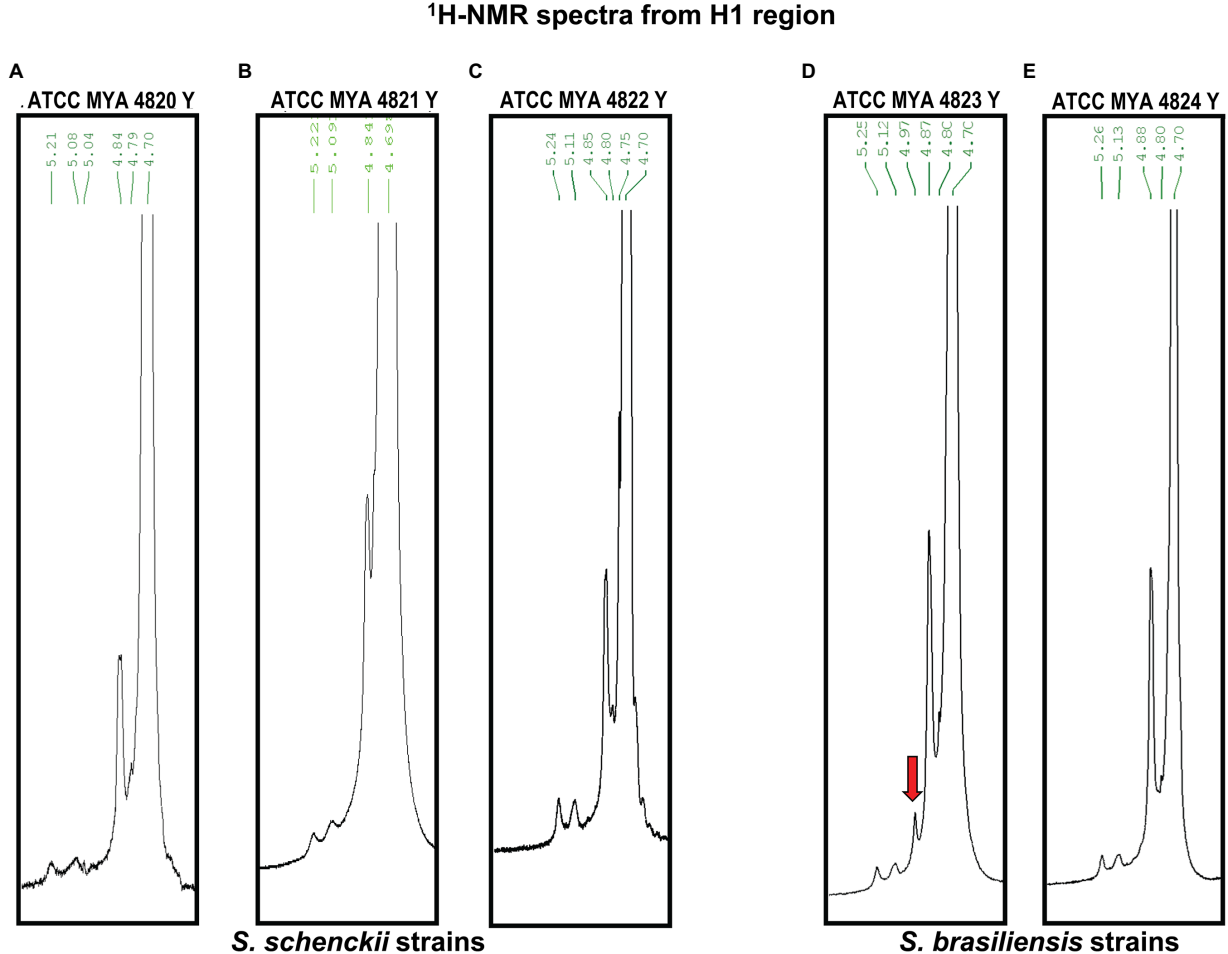
^1^H-NMR of the rhamnomannan fraction isolated from *S. schenckii* and *S. brasiliensis* yeast cells. The H1 region of the rhamnomannans of *S. schenckii* **(A–C)** and *S. brasiliensis* **(D,E)** is enlarged. The red arrow shows a unique signal for *S. brasiliensis* strain 4823 as was described recently (Lopes-Bezerra et al., 2018).

**Figure 4:**
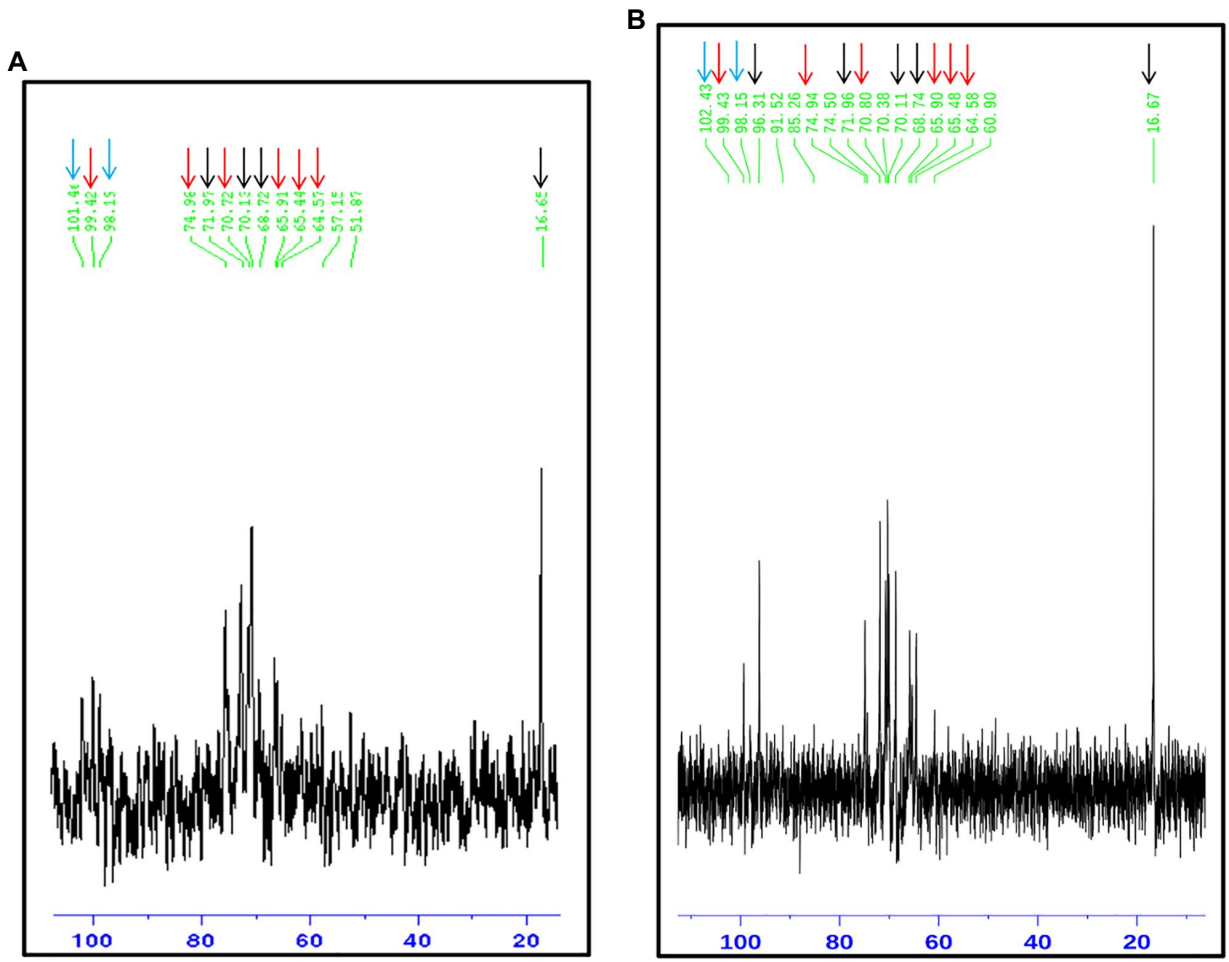
^13^C-NMR of the rhamnomannan fraction isolated from the strains presenting less virulence in yeast phase. *Sporothrix schenckii* 4822 (**A**) and *S. brasiliensis* 4824 (**B**) show the signals associated to type I rhamnomannan (red and black arrows), but also a signal corresponding to C-1 of α-L-Rhap nonreducing end unit of α-L-Rhap-(l, 2)-α—Rhap and 2,4-di-0-substituted α-D-mannopyranose units (Cian arrows; Travassos, 1985), which suggest longer side chains in the cell wall for these two strains.

The rhamnomannan characterization was followed by ^1^H-NMR and ^13^C-NMR. For all the cases, ^1^H-NMR spectra showed the presence of the methyl group that belongs to rhamnose (1.18–1.17ppm) and the signals corresponding to the proton linked to carbon 5 (3.32–4.10ppm) next to the methylene group from carbon six in the mannose ring (Figure 2B). The H1 region of the ^1^H-NMR spectra for all the strains under study is shown in Figure 3, presenting proton signals 5.21–5.26, 5.08–5.13, and 4.84–4.88ppm, which are characteristic of *Sporothrix* rhamnomannan, as previously reported (Travassos et al., 1973, 1974). Signals 5.08–5.12 and 5.21–5.25ppm are related to the presence of Rha(α1-4)GlcA(α1,2)Man(α1,2)Man-ol, as previously reported (Lopes-Alves et al., 1992). Also, the presence of a proton signal 4.97ppm previously reported as present in *S. brasiliensis* strain MYA 4823 (Lopes-Bezerra et al., 2018) is notoriously absent from the rhamnomannan of all the other strains (Figure 3D). The pattern of the ^13^C-NMR spectrum (Figure 2A) allowed us to determine how the rhamnose and mannan are linked in the rhamnomannan polymer. The rhamnomannan backbone is composed of mannose linked by α-1,6-glycosidic bonds and single units of rhamnose as side chains, which has been reported as characteristic of rhamnomannans isolated at 37°C from the *S. schenckii* yeast phase, first described as rhamnomannan type I (Figure 2A; Table 2; Travassos et al., 1973, 1974; Gorin et al., 1977). It is worth mentioning that the ^13^C-NMR spectra for the cell wall of *S. schenckii*, strain MYA4822, and *S. brasiliensis* MYA4824, showed unique signals at 98.15, 101.4, and 102.4ppm, associated with the C-1 of α-L-Rhap nonreducing end unit of α-L-Rhap-(l,2)-α-Rhap and 2,4-di-0-substituted α-D-mannopyranose units, which suggest longer side chains in the cell wall rhamnomannan for these two strains when compared to the other strains under study (Figure 4).

**Table 2:** ^13^C-NMR signals of *S. schenckii* and *S. brasiliensis* rhamnomannan, yeast phase.

Isolate	Structure	^13^CNMR – Signal, δ_c_ (70°C; ppm)						
		C1	C2	C3	C4	C5	C6	CH_3_
*S. schenckii* MYA-4820	α-L-Rhamnopyranose non-reducing end units	96.28	70.37	**N.R.**	71.94	68.74	-------	16.7
	3,6-di-O-substituted α-D-mannopyranose units	99.4	65.88	74.95	64.55	70.79	65.40	-------
*S. schenckii* MYA-4821	α-L-Rhamnopyranose non-reducing end units	96.28	70.36	**N.R.**	71.93	68.74	-------	16.7
	3,6-di-O-substituted α-D-mannopyranose units	99.44	65.88	74.92	64.55	70.82	65.397	-------
*S. schenckii* MYA-4822	α-L-Rhamnopyranose non-reducing end units	N.O.	70.13	**N.R.**	71.97	68.72	-------	16.7
	3,6-di-O-substituted α-D-mannopyranose units	99.4	65.91	74.98	64.57	70.72	65.44	-------
*S. brasiliensis* MYA-4823	α-L-Rhamnopyranose nonreducing end units	96.39	70.17	**N.R.**	72.05	68.8	-------	16.7
	3,6-di-O-substituted α-D-mannopyranose units	99.54	66.04	75.08	64.65	70.92	65.57	-------
*S. brasiliensis* MYA-4824	α-L-Rhamnopyranose non-reducing end units	96.31	70.11	**N.R.**	71.96	68.7	-------	16.7
	3,6-di-O-substituted α-D-mannopyranose units	99.43	65.9	74.94	64.58	70.8	65.48	-------
Gorin et al., 1977	α-L-Rhamnopyranose non-reducing end units	**98.3**	**72–71.9**	**N.R.**	**73.6**	**70.8**	-------	**18.4**
	3,6-di-O-substituted α-D-mannopyranose units	**101.1**	**67.6**	**76.6**	**66.3**	**72.4**	**67.3**	-------

N.O., not observed; N.R., no registered. Bold values correspond to values previously reported.

### Polysaccharide Quantification in the Cell Wall Fractions

**Figure 5:**
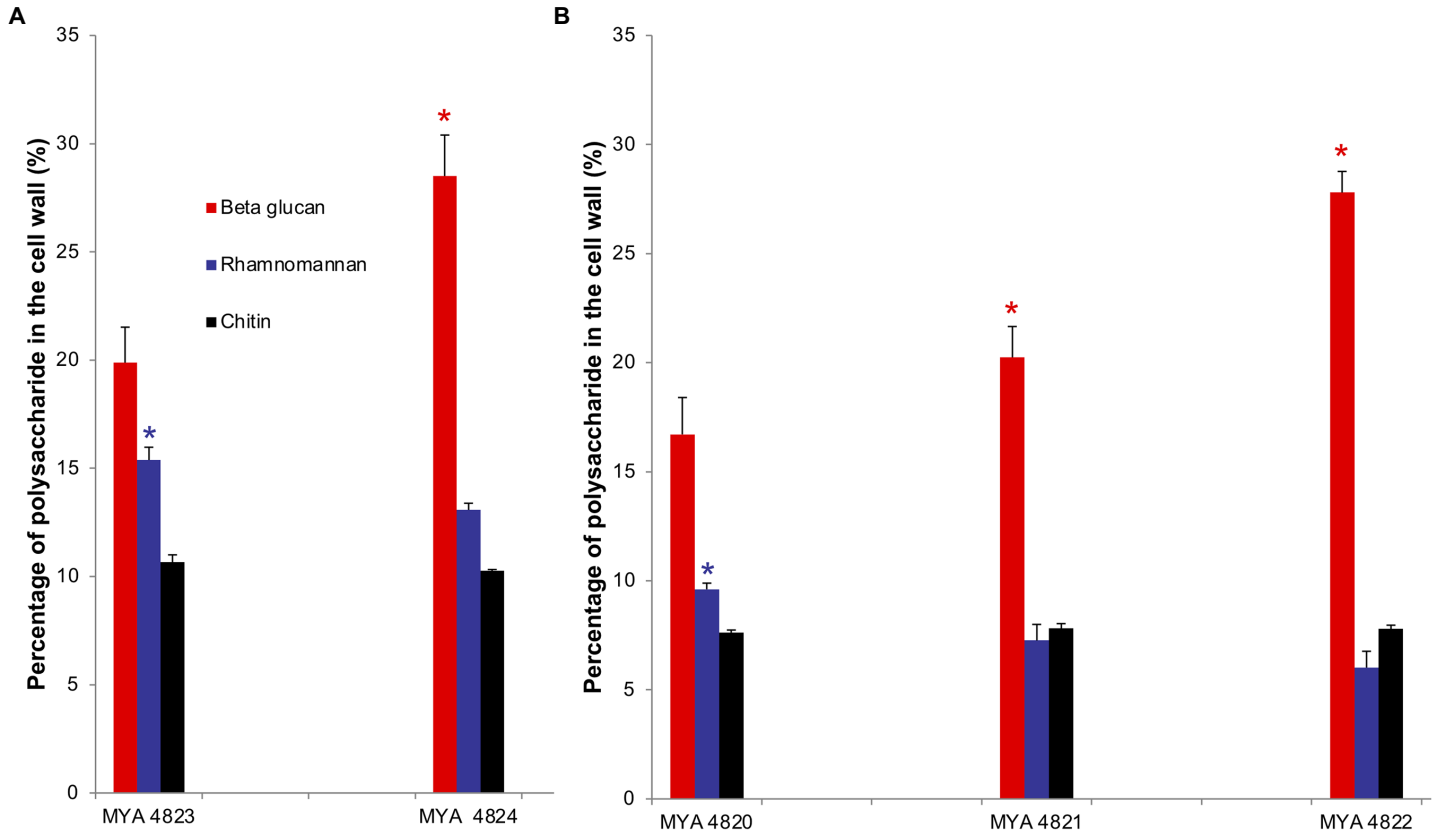
Comparison of polysaccharides represented as percentage in the cell wall of *S. brasiliensis* (**A**) and *S. schenckii* (**B**) strains, yeast phase. Percentage of polysaccharides are represented in colored bars: β glucan (red), rhamnomannan (blue) and chitin (black). ^*^Tukey Honestly Significant Difference (HSD) *post hoc* test was used for intra and inter species comparative analyses. Value of *p*<0.05. Quantification of cell wall components was made by triplicate.

**Figure 6:**
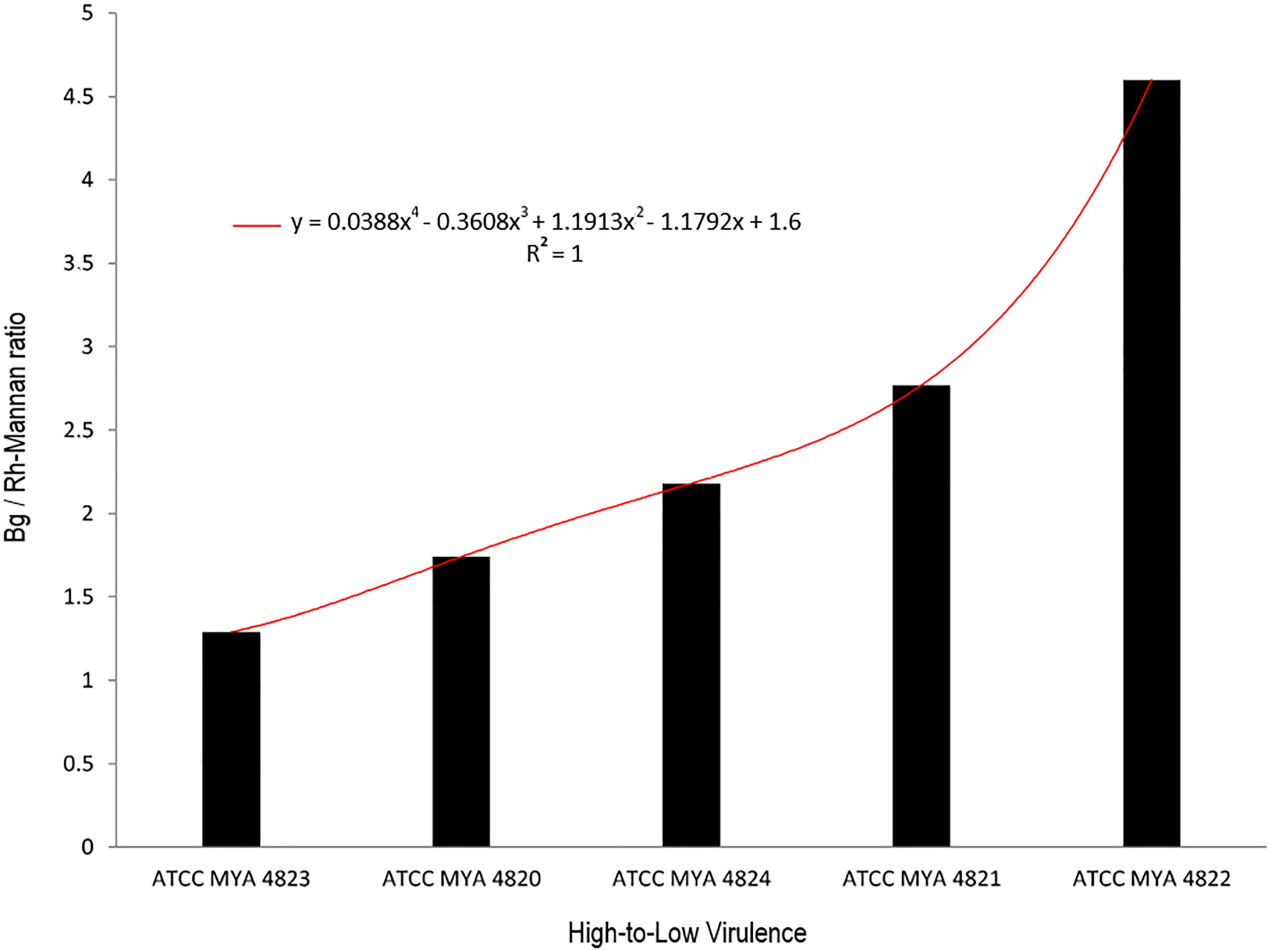
Relation between β glucan vs. rhamnomannan ratio with strain virulence. Black columns represent the ratio of β glucan to rhamnomannan present in the cell wall. Strains are arranged from higher to the lower virulence reported. The red curve represents the polynomial curve of relationship, mathematically represented by an ascendant curve with an *R*^2^=1 with the polynomial function *y*=0.0388*x*^4^–0.3608*x*^3^+1.1913*x*^2^–1.1792*x*+1.6. Bg, β-glucan; Rh-Mannan, rhamnomannan.

For polysaccharide quantification, the fractions obtained by alkali and acid fractionation were further analyzed by colorimetric techniques as described in the methods section. Table 3 shows the relative cell wall polysaccharides content for *S. schenckii* and *S. brasiliensis* strains. The polysaccharide analysis for the cell walls of all strains in the yeast phase, showed a higher chitin content (around 27%) for the two *S. brasiliensis* strains, when compared to the *S. schenckii* strains (Table 3; Figure 5), as previously reported (Lopes-Bezerra et al., 2018). A difference was evident for the cell wall β-glucan relative content (around 28% more β-glucan) of the lower virulent *S. schenckii* MYA 4822 and *S. brasiliensis* MYA 4824 strains when compared to the higher virulent strains (Table 3; Figure 5). Rhamnomannan relative contents were higher for both *S. brasiliensis* strains analyzed, when compared to the *S. schenckii* strains (up to 38% more rhamnomannan). Also, a higher rhamnomannan relative content could be observed in the more virulent *S. schenckii* MYA 4820 strain, when compared with the non-virulent *S. schenckii* MYA 4822 strain (over 30% higher; Table 3; Figure 5). The relationship between the level of virulence reported and the β-glucan/rhamnomannan cell wall ratio can be represented mathematically by an ascendant curve, with an *R*^2^=1 for the polynomial function *y*=0.0388*x*^4^–0.3608*x*^3^+1.1913*x*^2^–1.1792*x*+1.6 (Figure 6), which shows an inverse relationship between the reported virulence and a higher ratio of cell wall β-glucans/rhamnomannan content.

**Table 3:** Cell wall polysaccharide content comparison of the *Y* phase of the *S. schenckii* and *S. brasiliensis* strains under study.

Strain	*S. schenckii* MYA 4820	*S. schenckii* MYA 4821	*S. schenckii* MYA 4822	*S. brasiliensis* MYA 4823	*S. brasiliensis* MYA 4824
Beta glucan	16.7±1.7	20.2±1.4	27.8±1.0	19.9±1.6	28.5±1.9
Rhamnomannan	9.6±0.3	7.3±0.7	6.0±0.8	15.4±0.6	13.1±0.3
Rhamnose	6.5±0.6	5.4±0.1	3.7±0.1	10.8±0.1	10.8±0.2
Chitin	7.6±0.1	7.8±0.2	7.8±0.2	10.7±0.4	10.3±0.1

Tukey Honestly Significant Difference (HSD) *post hoc* test was used for intra and inter species comparative analyses. Value of *p*<0.05. Quantification of cell wall components were made by triplicate. SEM is shown.

**Figure 7:**
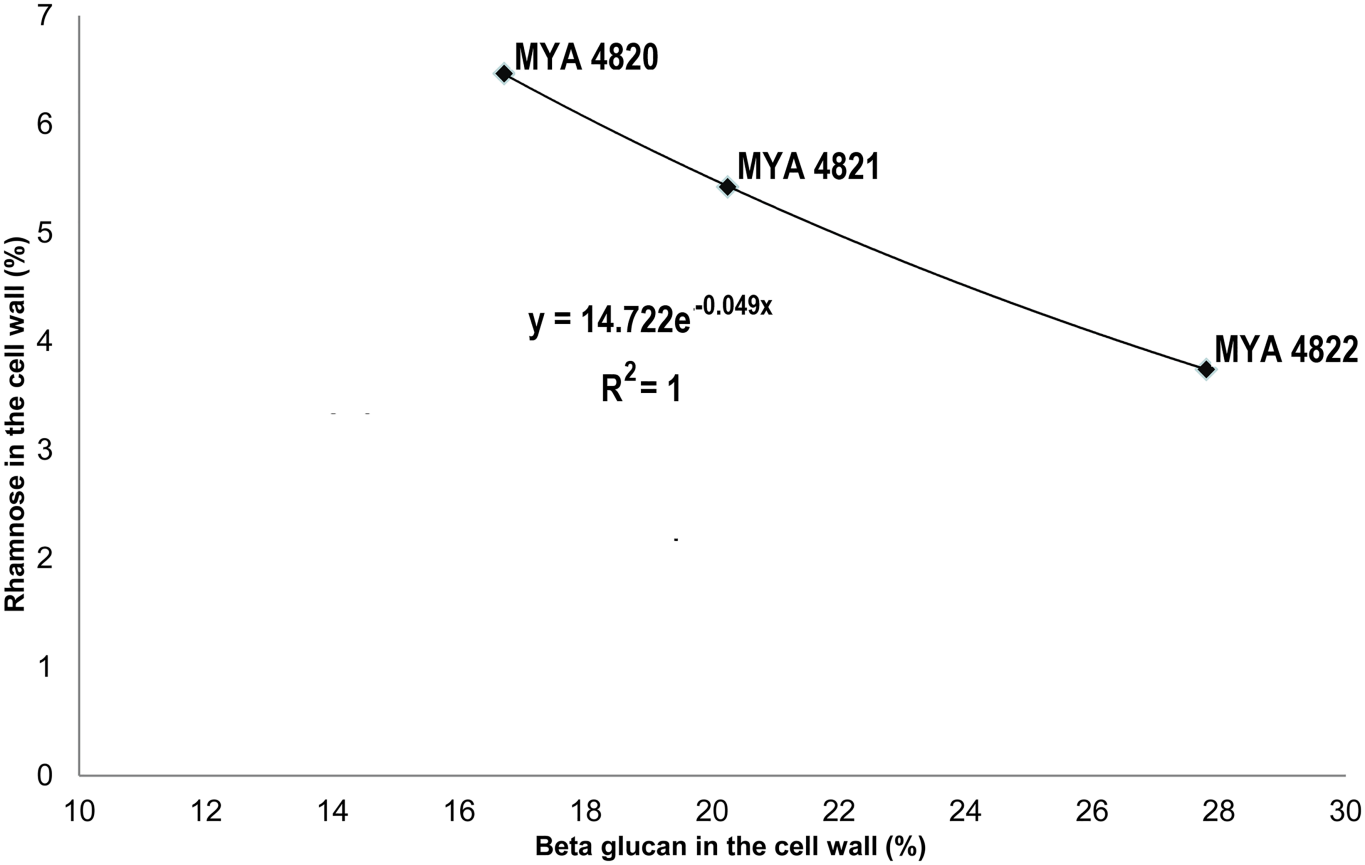
A mathematical model for the Rhamnose/β-glucan composition as expression of virulence. With an increase in reported virulence, the rhamnose proportion rise and β-glucan decreases. This observation fit to an exponential curve with an *R*^2^=1, that could be expressed as a linear equation: *ρ*=−0.049(*β*)+2.7, where *β* represents the β-glucan composition and *ρ*=Ln(Rha), where Rha is the rhamnose cell wall percentage. This model might be useful to predict the virulence level employing the β-glucan and rhamnose percentage ratio. This mathematical expression infers the highest rhamnose percentage to 15% (*β*=0) and for the lowest rhamnose percentage (1%) *β*=55.1%.

Rhamnose residues from PRM are known to be the main antigenic epitopes found on the *S. schenckii* cell surface (Fernandes et al., 1999). Here, the rhamnose content in *S. brasiliensis* strains was 40% higher compared to *S. schenckii* strains (Table 3). When comparing only the *S. schenckii* strains, the cell wall rhamnose content shows differences from high-to-low virulence for strains MYA 4820, MYA 4821, and MYA 4822 (Table 3; Figure 5). This observation fits the exponential curve: *y*=14.722e^−0.049x^, with an *R*^2^=1, that can be mathematically expressed as the linear equation: *ρ*=−0.049 (*β*)+2.7, where *β* represents the cell wall β-glucan content expressed as percentage and *ρ* represent the Ln_(Rha)_, where Rha is the rhamnose cell wall content represented as a percentage (Figure 7). No significant differences were observed for the cell wall β-glucans among strains, except for *S. schenckii* MYA 4821, which had 20% more β-glucans than the rest of the analyzed strains.

### Chitin Exposure on the Yeast Cell Wall

**Figure 8:**
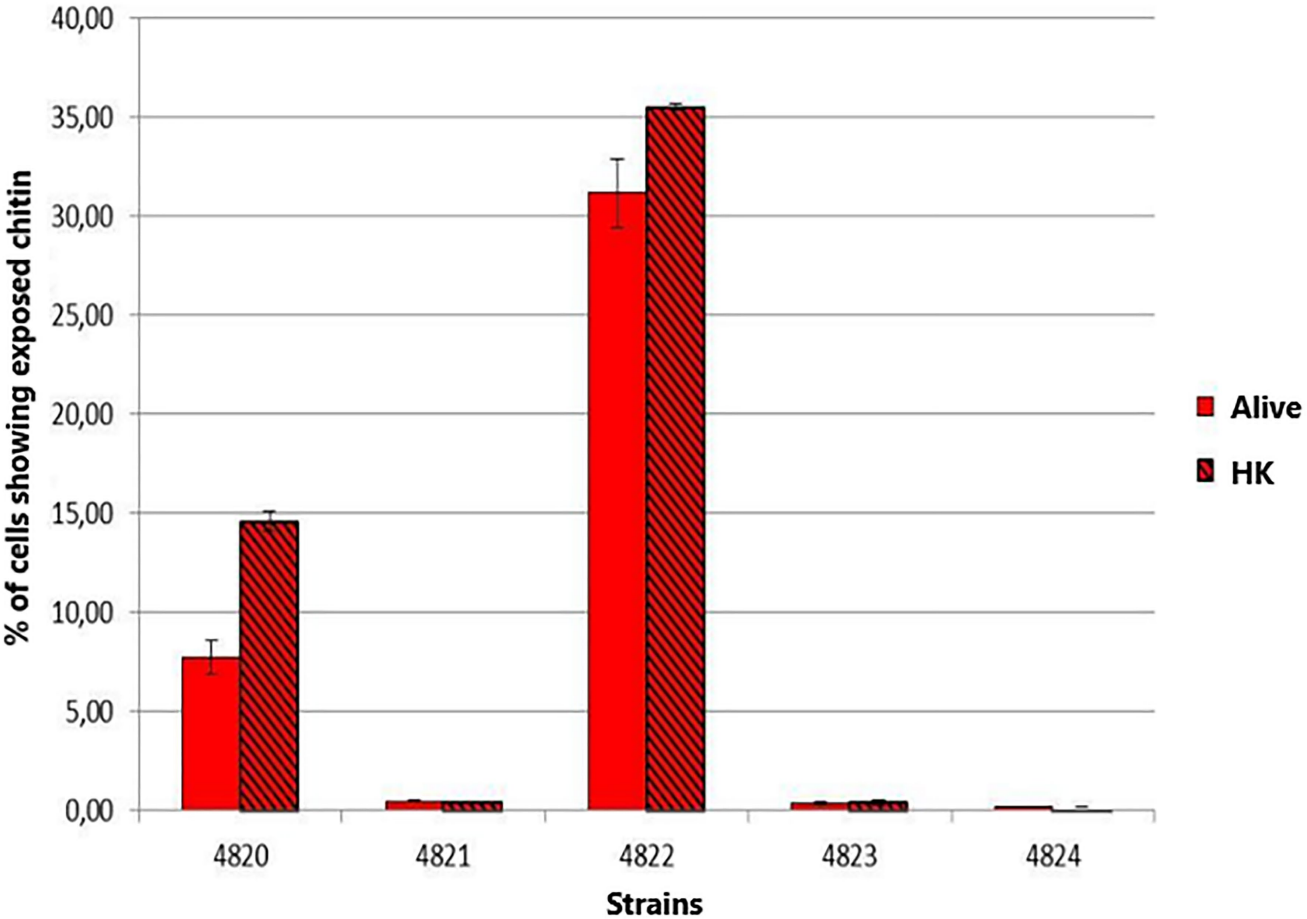
Comparison of the chitin exposure on the yeast cells of *S. schenckii* and *S. brasiliensis* grown in Brain Heart Infusion (BHI) broth. The smooth bars represent the exposure of chitin under normal and live cell (Alive). The bars with frames represent the percentage of exposed chitin after the cells were heat inactivated (HK).

Chitin exposure on the cell wall for the five strains under study was determined in BHI-grown yeast cells. The highest chitin exposure on the cell wall was found for *S. schenckii* strain MYA 4822 (Figure 8), followed by *S. schenckii* strain MYA 4820. The lowest cell wall chitin exposure was observed for *S. schenckii* MYA 4821, and the two *S. brasiliensis* strains MYA 4823 and MYA 4824, all of them reported as presenting higher virulence patterns (Nascimento et al., 2008; Castro et al., 2013).

## Discussion

The cell wall is the first point of contact with the host upon infection and colonization; understanding its composition allow unveiling specific mechanisms triggered by PAMPs and their corresponding PRRs (Gow et al., 2017). Recently, it was reported that carbon or nitrogen limitation during growth of yeast cells of *S. brasiliensis* and *S. schenckii* resulted in a reduced virulence, and the mechanism is related to affect the cell wall composition, where an increase in cell wall β-glucan, and a reduction of rhamnose and mannose was observed (Lozoya-Pérez et al., 2020). Also, the virulence-reduced strains showed a higher exposure of β-glucan, leading to an increase in the uptake of the fungus by hemocytes of *Galleria mellonella* (Lozoya-Pérez et al., 2020).

In the present work, we compared the cell relative composition of the polysaccharides of the pathogenic yeast morphotype of five *Sporothrix* strains, of which three were *S. schenckii* and two *S. brasiliensis* strains, with differences in virulence levels reported in a murine model (Table 1; Nascimento et al., 2008; Castro et al., 2013). To normalize the comparison, all the strains were grown under identical conditions in BHI broth, a widely used culture medium for *Sporothrix* spp. (Kong et al., 2006; Brito et al., 2007; Teixeira et al., 2009; Della Terra et al., 2017; De Almeida et al., 2018).

As previously reported, the main polysaccharides present in the cell wall of both *S. schenckii* and *S. brasiliensis* strains were: β-glucan, as major cell wall polysaccharide, followed by rhamnomannan and chitin (Table 3; Figure 5; Lopes-Bezerra et al., 2018; Lozoya-Pérez et al., 2020). A higher cell wall chitin content was observed in the cell wall of *S. brasiliensis* strains compared to *S. schenckii* strains, which also have been previously reported (Lopes-Bezerra et al., 2018). However, when comparing the cell wall polysaccharide composition of the five *Sporothrix* spp. strains, a pattern appeared to emerge, with higher β-glucans and lower rhamnomannan levels in cell wall contents present in the previously reported non-virulent or low virulent strains (*S. schenckii* MYA 4822 and MYA 4821 and *S. brasiliensis* MYA 4824). In contrast, lower β-glucan and higher rhamnomannan levels in cell wall content were shown in those strains for which higher virulence have been reported, regardless of the species (*S. brasiliensis* MYA 4823 and *S. schenckii* MYA 4820; Table 3; Figure 5). Therefore, the β-glucan/rhamnomannan cell wall ratio can be mathematically represented by a polynomial function showing an inverse relationship to the virulence increase (Figure 6). Then, we focused on *S. schenckii*, for which we had strains with three different levels of virulence reported (Table 1) and noticed that cell wall rhamnose content increased, while the cell wall β-glucan content decreased when compared from the less to the highest reported virulence phenotype (Table 3; Figure 5). This observation can be mathematically expressed as a linear equation (Figure 7), which extrapolates the highest virulence for *S. schenckii* strains when the rhamnose percentage in the cell wall reaches 15% and the β-glucans cell wall content is 0%, and the lowest virulence when the rhamnose percentage is 1% and the β-glucan content is 55.1% (intersection points on the *y* and *x* axis of the linear curve, respectively, Figure 7).

Recently, a bilayered cell wall model based on experimental data was proposed for *S. schenckii* and *S. brasiliensis* yeast cells (Lopes-Bezerra et al., 2018), which positioned the structural and more immunogenic chitin and β-glucans at the inner-most layer, and the PRM as an outermost layer covering the former.

The structural cell wall glycoconjugates, β-1-3 and β-1-6-glucans, as well as chitin, are found in pathogenic fungal species as involved in the innate immune response as PAMPs, so the exposure of β-glucans and chitin on the fungal surface favors their binding to their corresponding PRRs presented on the host cells surface, allowing the uptake of the microorganism and/or triggering the secretion of specific cytokines (Hernández-Chávez et al., 2017). A *Sporothrix* spp. strain with a higher β-glucans/rhamnomannan ratio might favor the exposition of the immunogenic β-glucans to the host immune system, triggering its response before the infection can be established, therefore presenting a lower level of virulence. Indeed, Lozoya-Pérez et al. (2020) recently reported that a higher β-glucan exposure is in close relation with a lower virulence phenotype in *Sporothrix* spp. To determine whether chitin also might be playing a role in the differences in virulence levels, chitin exposition was measured in the Y pathogenic phase for the five *Sporothrix* strains. Only the non-virulent *S. schenckii* MYA 4822 presented a high chitin exposition on its cell surface under the growth conditions used in the present study (Figure 8), which together with the high β-glucans/rhamnomannan ratio, builds up evidence for the involvement in the non-virulence phenotype reported, and by triggering the host immune system more efficiently.

A conserved general structure of the cell wall polysaccharides for all *Sporothrix* strains in their yeast phase was evidenced by the IR spectra analyzed. However, some differences were observed when the rhamnomannans from the cell walls of the five *Sporothrix* strains were characterized by ^1^H-NMR and ^13^C-NMR. Although a general pattern for both spectra was apparent for all the five strains studied (Figure 2), a closer inspection of the ^13^C-NMR spectra allowed us to identify unique signals for the cell wall rhamnomannan of the non-virulent and low virulent *S. schenckii* MYA 4822 and *S. brasiliensis* MYA 4824, respectively at 98.15, 101.4, and 102.4, associated with the C-1 of α-L-Rhap non-reducing end unit of α-L-Rhap-(l,2)-α-Rhap and 2,4-di-0-substituted α-D-mannopyranose units, suggesting longer side chains in the cell wall rhamnomannan for these two strains. Methylation analyses of the rhamnomannan present in the reportedly least virulent strains and comparison with the higher virulent strains would provide further insight into such differences. Also, the comparison of the ^1^H-NMR spectra for the rhamnomannan of all the strains studied, confirmed a previous report, showing the presence of a 4.97ppm signal only for the *S. brasiliensis* MYA 4823, which has been reported as a high virulent strain (Castro et al., 2013). The analysis of virulent and non-virulent strains of the *Sporothrix* genus, suggests that the rhamnomannans of the cell wall determines the exposure of chitin and β-glucans, which ultimately triggers a strong immune response that explains the resulting virulence phenotype. To overcome the limitations of the present work and to either strengthen or discard the mathematical model of virulence here proposed, a broader study including more strains, testing their virulence in a single mathematical model of virulence for sporotrichosis, and exploring the alterations in cell wall composition from strains cultured in different media and their possible impacts on virulence would be necessary, and will definitely either reinforce or discard this model to assess virulence, specifically for the *Sporthrix* genus.

## Data Availability Statement

The original contributions presented in the study are included in the article/supplementary material, further inquiries can be directed to the corresponding author.

## Author Contributions

HV-D, LL-B, and GN-V conceived and designed the experiments. HV-D, LB, ÁA-A, BF, and NL-P performed the experiments. HV-D, GN-V, LL-B, and HM-M analyzed the data. HV-D and GN-V wrote the paper. All authors contributed to the article and approved the submitted version.

## Funding

HV-D was supported by Instituto Venezolano de Investigaciones Cientificas, Venezuela (Project 112). GN-V was supported by CONACYT-Mexico (Ref. CF-2019-170701). HM-M was supported by CONACYT-Mexico (Ref. FC 2015-02-834). Flow cytometry analysis was supported by CONACYT (grants 3013–205744 and 2019–300286).

## Conflict of Interest

The authors declare that the research was conducted in the absence of any commercial or financial relationships that could be construed as a potential conflict of interest.

## Publisher’s Note

All claims expressed in this article are solely those of the authors and do not necessarily represent those of their affiliated organizations, or those of the publisher, the editors and the reviewers. Any product that may be evaluated in this article, or claim that may be made by its manufacturer, is not guaranteed or endorsed by the publisher.
